# Fibrosis in metastatic lymph nodes is clinically correlated to poor prognosis in colorectal cancer

**DOI:** 10.18632/oncotarget.25636

**Published:** 2018-07-03

**Authors:** Daiji Ikuta, Toru Miyake, Tomoharu Shimizu, Hiromichi Sonoda, Ken-Ichi Mukaisho, Aya Tokuda, Tomoyuki Ueki, Hiroyuki Sugihara, Masaji Tani

**Affiliations:** ^1^ Department of Surgery, Shiga University of Medical Science, Shiga, Japan; ^2^ Department of Molecular and diagnostic Pathology, Shiga University of Medical Science, Shiga, Japan

**Keywords:** cancer-associated fibroblast, colorectal cancer, fibroblastic reticular cell, fibrosis, lymph node metastasis

## Abstract

**Background:**

Tumor microenvironment including fibrosis has a pivotal role in cancer growth and distant metastasis. Fibrosis is a known risk factor for carcinogenesis, but its biological role in disease invasion and metastasis in colorectal cancer (CRC) remains unclear. In particular, there is no report on how fibrosis of metastatic lymph nodes (MLNs) in CRC contributes to prognosis.

**Methods:**

We reviewed 94 colorectal adenocarcinoma patients with MLNs who underwent colectomy. Both the primary tumors and MLNs were analyzed for alpha-smooth muscle actin (α-SMA) expression and collagen deposition.

**Results:**

Higher α-SMA expression and collagen deposition in MLNs were associated with significantly shorter relapse-free survival and overall survival in CRC patients. α-SMA expression in MLNs (HR, 1.53; *p =* 0.034) was independent predictive factor of overall survival in multivariate Cox proportional hazards regression analysis of clinicopathological factors. In the Stage III patient subgroup, α-SMA expression in MLNs was a strong prognostic marker (HR, 3.01; *p =* 0.006). On the other hand, higher α-SMA expression and collagen deposition in primary tumors were associated with short overall survival, but they were not significant factors in multivariate Cox regression analyses. In MLNs, the podoplanin signals co-localized with α-SMA expression and were confirmed by the dual immunofluorescence staining, implying that the MLN stromal cells were fibroblastic reticular cells.

**Conclusion:**

Both high collagen deposition and high α-SMA expression in MLNs predicted poor prognosis in CRC.

## INTRODUCTION

Colorectal cancer (CRC) is the third most common malignancy worldwide [1]. Advanced CRC metastasizes to many organs such as the lymph nodes (LNs), peritoneum, lungs, and the liver. Metastasis is implicated in 90% of all CRC-related deaths. Although advances in chemotherapy and targeted drug therapies have ensured a prolonged survival in CRC patients, the 5-year survival rate of patients with Stage III disease and LN metastasis is from 55% to 73% for colon cancer and from 45% to 69% for rectal cancer, while that of patients with Stage IV disease and distant metastasis is less than 20% [2, 3].

LNs are immune organs that respond to peripheral infections through the T cell-dependent paracortical areas. Antigen-presenting dendritic cells within the T cell-dependent paracortical areas then prime the naive T lymphocytes. Then, through the B cells-containing cortical germinal follicles, the naive B lymphocytes produce antibodies in response to the antigen [4]. LN metastasis occurs when cancer cells detach from the primary tumor (PT), enter the lymphatics, and are subsequently transported to regional LNs. LN metastasis is an invaluable prognostic factor in almost all cancer types. Mesenteric LNs are the major metastatic sites for CRC, and metastatic LN (MLN) number and position are used in cancer staging. Furthermore, metastasis of LNs prognosticates survival rate in CRC [5–7]. Chronic inflammation caused by cancer cells stimulates surrounding cells including fibroblasts, and activated fibroblasts with alpha-smooth muscle actin (α-SMA) expression produce extracellular matrix including collagen, finally causing tissue fibrosis.

Fibroblasts populate stromal tissue in the tumor microenvironment, and are key mediators of fibrosis. Organ fibrosis is a risk factor for carcinogenesis in the cirrhotic liver, lungs, kidneys, and skin [8–11]. Fibroblasts in cancer tissues, known as cancer-associated fibroblasts (CAFs), cause tissue remodeling and reconstitution to promote invasion and metastasis via extracellular matrix, growth factors, and protease production [12]. This gamut of molecular events is termed stromal reaction (SR), but its role in tumor progression and invasiveness is still controversial. Several clinical studies have reported that a strong SR in CRC is related to poor prognosis [13–15]. However, evidence exists to suggest a positive correlation between SR and better prognosis [16].

The SR of MLNs has not received adequate attention, with only a few reports, pertaining to lung cancer, having been published [17, 18]. In particular, there was no conclusive report on how the SR status of MLNs in CRC contributes to prognosis. Our aim was to elucidate the clinical impact of fibrosis of PTs and MLNs in CRC.

## RESULTS

The median age of the patients was 66 (interquartile range, 60 to 72) years, and the group consisted largely of males, 59 (62.8%). The median follow-up time for all patients was 59.9 (interquartile range, 38.9 to 72.3) months. Among the patients, 32 deaths and 23 relapse occurred.

**Figure 1:**
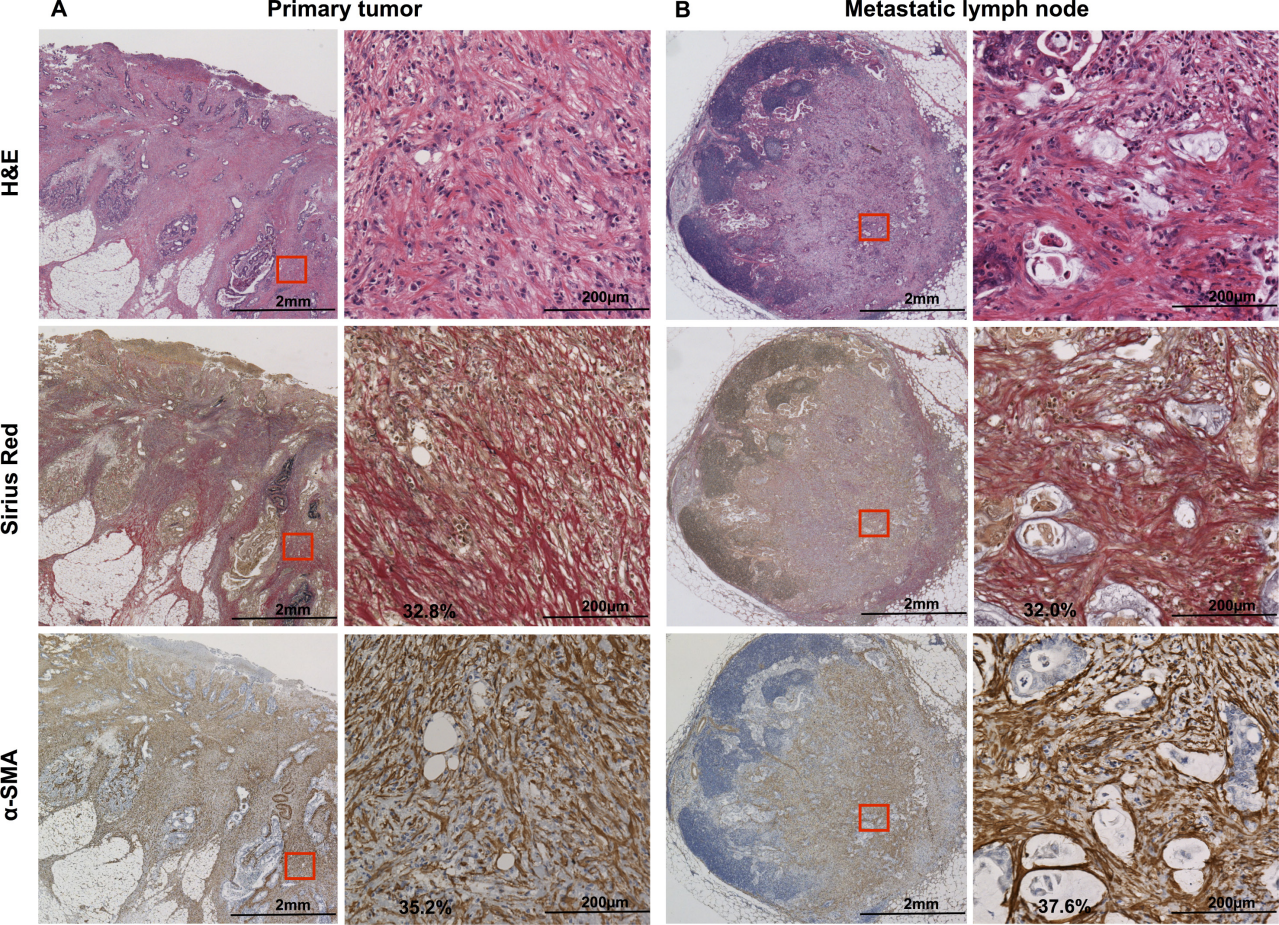
H&E, Sirius Red, and α-SMA staining of (**A**) primary tumor and (**B**) metastatic lymph node. Sirius Red stains type I and III collagen in red. α-SMA is expressed in mesenchymal cells and stains brown upon immunohistochemistry. A field of view of 200× microscopic images (demarcated in red on the left) is shown on the right side, and was used for the statistical analysis. H&E, hematoxylin and eosin; α-SMA: α-smooth muscle actin.

The representative images of the PT (Figure 1A) and the MLN (Figure 1B) based on the evaluated PT and MLN sections by hematoxylin and eosin (H&E), depict that both tissue types showed α-SMA expression and collagen deposition. Furthermore, in order to characterize the fibroblast, we performed immunohistochemistry (IHC) staining of fibronectin, fibroblast activation protein (FAP), and CD31 (Supplementary Figure 1).

We compared the diameters of 147 MLNs in various patients and the staining rate of α-SMA or collagen, and showed that there was no correlation between the diameter and the degree of fibrosis in MLN; Pearson’s correlation coefficients were 0.171 and 0.119, respectively.

In a subset of 41 patients with multiple MLNs, a strong positive correlation was found between the α-SMA expression rate of the largest-diameter MLN and the average α-SMA expression rate of all MLNs in the same patient (Pearson’s correlation coefficient: 0.859; Supplementary Figure 2A). A similar correlation was also observed for collagen (Pearson’s correlation coefficient: 0.803; Supplementary Figure 2B). Therefore, we selected the largest MLN for analyses. Thus, it was not necessary to measure the α-SMA expression rate for all MLNs in clinical practice; hence, the data collection was more convenient. These results suggest that the degree of fibrosis of MLNs was not dependent on MLN diameter but by the nature of metastatic tumor.

### Collagen deposition in MLNs in CRC is associated with poor prognosis

Collagen deposition rate in the largest MLNs differed widely, ranging from 1.0% to 58.1% (Supplementary Figure 3A) while the positive range of PTs was from 10.3% to 43.2% (Supplementary Figure 3B). Collagen deposition was significantly higher in MLNs than in non-MLNs (MLN: 28.5 **±** 15.0%; non-MLN: 2.1 ± 1.7%; *P <* 0.001). Similarly, collagen deposition rate at the invasive front of PTs was significantly higher than that in normal tissue (PT: 20.9 **±** 6.8%; normal tissue: 2.9 ± 1.3%; *P <* 0.001). A weak correlation occurred between collagen deposition rate of PTs and MLNs (Pearson’s correlation coefficient: 0.324).

In Table 1, we showed the clinicopathological factors classified by high and low collagen deposition or α-SMA expression in MLNs. Significant differences occurred with higher collagen deposition (in MLN) in pathological tumor stage (*p =* 0.032), pathological node stage (*p =* 0.031), liver metastasis (*p =* 0.048), pathological stage (*p =* 0.013), and recurrence (*p <* 0.001).

**Table 1:** Clinicopathological factors classified by collagen deposition and α-SMA expression in metastatic lymph nodes

Factors		*N*	Collagen		*p*-value	α-SMA		*p*-value
			High (*N* = 39)	Low (*N* = 55)		High (*N* = 40)	Low (*N* = 54)	
Age		94	66 (60–73)	66 (61–72)	0.854	66 (60–74)	66 (60–72)	0.643
Gender	Male	59	25	34	0.498	25	34	0.566
	Female	35	14	21		15	20	
Preoperative CEA		94	10.3 (5.8–32.8)	5.0 (3.3–14.7)	0.025^*^	8.6 (3.9–19.5)	7.1 (3.1–18.2)	0.479
Tumor site	Right^**1)**^	28	8	20	0.076	10	18	0.260
	Left^**2)**^	66	31	35		30	36	
Pathological tumor stage	T2	11	1	10	0.032^*^	4	7	0.313
	T3	64	27	37		25	39	
	T4	19	11	8		11	8	
Pathological node stage	N1	62	21	41	0.031^*^	24	38	0.203
	N2	32	18	14		16	16	
Histological type	Well^**3)**^	86	36	50	0.561	35	51	0.206
	Poor^**4)**^	8	3	5		5	3	
Lymphatic permeation	ly0,1	58	22	36	0.250	24	34	0.468
	ly2,3	36	17	19		16	20	
Vascular invasion	v0,1	49	18	31	0.222	19	30	0.286
	v2,3	45	21	24		21	24	
Liver metastasis	H(–)	72	26	46	0.048^*^	27	45	0.062
	H(+)	22	13	9		13	9	
Peritoneal dissemination	P(–)	89	36	53	0.340	39	50	0.288
	P(+)	5	3	2		1	4	
Pathological Stage	Stage III	66	22	44	0.013^*^	25	41	0.119
	Stage IV	28	17	11		15	13	
Recurrence	Liver	8	7	1	<0.001^*^	7	1	<0.001^*^
	Lung	8	6	2		6	2	
	Local recurrence	7	4	3		5	2	
	No recurrence	50	12	38		13	37	
Postoperative chemotherapy	With oxaliplatin	47	24	23	0.306	22	25	0.530
	Without oxaliplatin	29	9	20		10	19	
	No chemotherapy	18	6	12		8	10	

Associations between categorical variables were analyzed using either the χ^2^ test or the Fisher’s exact test. Age and preoperative CEA were analyzed using Mann–Whitney *U* test and expressed as the median value (interquartile range). 1) Right includes from cecum to transverse colon. 2) Left includes from descending colon to rectum below the peritoneal reflection. 3) Well includes 24 cases of well-differentiated adenocarcinoma and 62 cases of moderately differentiated adenocarcinoma. 4) Poor includes 4 cases of poorly differentiated adenocarcinoma, 1 case of signet ring cell carcinoma and 3 cases of mucinous carcinoma. ^*^Statistically significant. Abbreviations: α-SMA: α-smooth muscle actin; CEA: Carcinoembryonic antigen.

**Figure 2:**
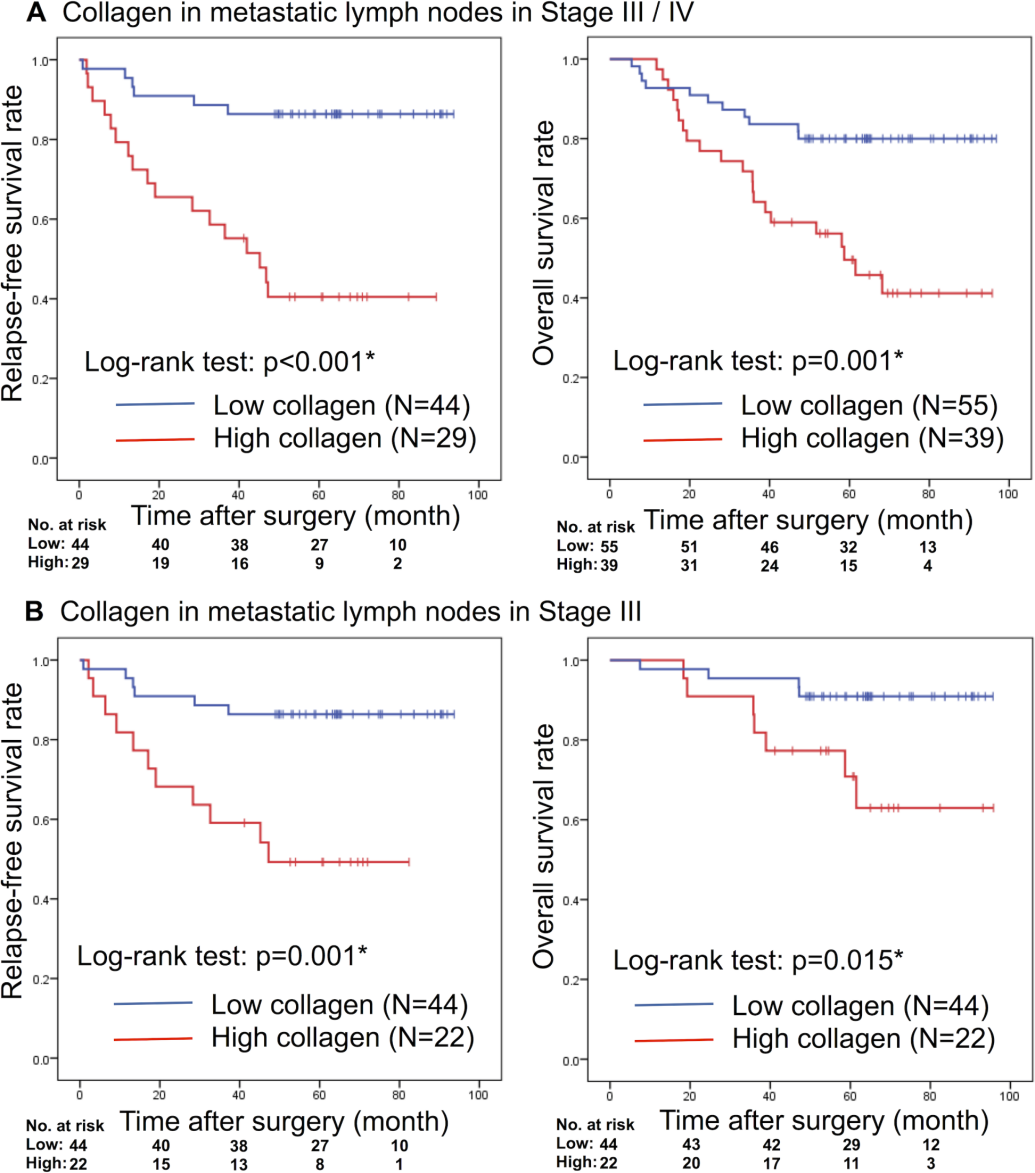
Kaplan–Meier analyses of RFS and OS rates based on the collagen deposition rate of metastatic lymph nodes in (**A**) Stages III/IV disease or (**B**) only Stage III disease. RFS: relapse-free survival; OS: overall survival. ^*^Statistically significant.

Higher collagen content (“High”) was indicative of higher recurrence rate (high: 58.6%; low: 13.6%; χ^2^ test, *p <* 0.001; Table 1). As shown in Figure 2A, both relapse-free survival (RFS) and overall survival (OS) of the “High” collagen group were significantly shorter than those of the “Low” collagen group (RFS, log-rank *p <* 0.001; OS, log-rank *p =* 0.001). As Figure 2B shows, the subgroup analysis of Stage III showed that the prognosis and the recurrence rate of the “High” group were significantly shorter than those of the “Low” group (RFS, log-rank *p =* 0.001; OS, log-rank *p =* 0.015).

At the invasive front of PTs (all patients), high collagen deposition correlated with shorter RFS (log-rank *p =* 0.009) and OS (log-rank *p =* 0.002; Supplementary Figure 4A). Subgroup analysis showed that in Stage III patients, high collagen deposition of MLNs correlated with poor prognosis, but collagen deposition at the invasive front of the PTs did not significantly correlate with OS or RFS (Supplementary Figure 4B).

### α-SMA expression of MLNs in CRC is related to RFS and OS

We analyzed α-SMA expression in PTs and MLNs. The largest MLNs ranged from 13.5% to 49.6% positivity (Supplementary Figure 3C) while the positive range of PTs was from 10.3% to 43.2% (Supplementary Figure 3D) for α-SMA expression. Similar to the collagen deposition, α-SMA expression was significantly higher in MLNs vs non-MLN (29.5 **±** 8.6% vs 1.7 ± 1.9%, respectively; *p <* 0.001) and PTs vs normal tissue (26.6 **± 7.**7 vs 1.2 ± 1.1%, respectively; *p <* 0.001). There was no correlation between α-SMA expression rate of PTs and MLNs (Pearson’s correlation coefficient: 0.134).

**Figure 3:**
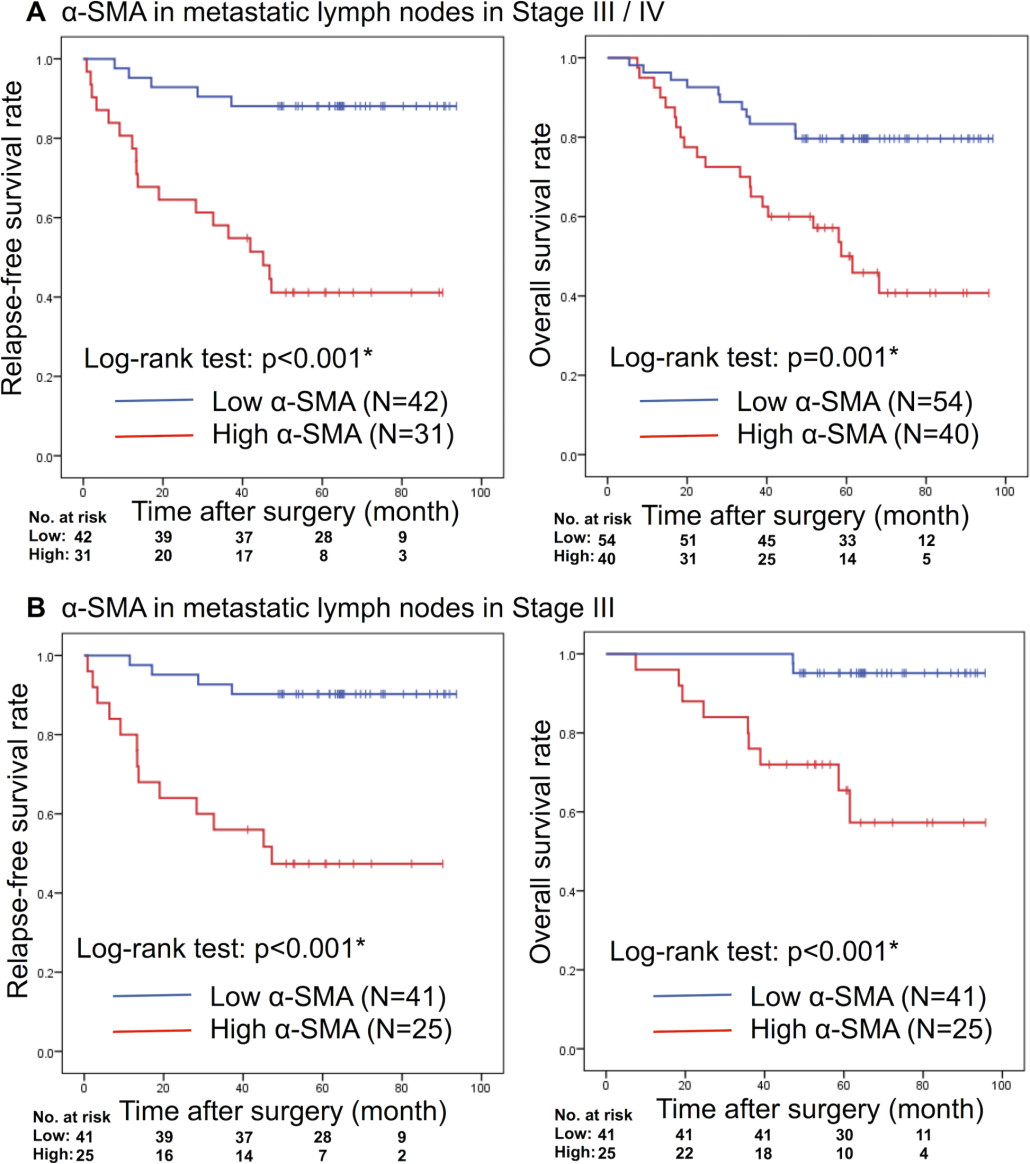
Kaplan–Meier analyses of RFS and OS rate based on α-SMA expression rate of metastatic lymph nodes in (**A)** Stages III/ IV disease or (**B**) only Stage III disease. RFS: relapse-free survival; OS: overall survival; α-SMA: α-smooth muscle actin. ^*^Statistically significant.

Clinicopathological factors were analyzed against α-SMA expression (Table 1), and revealed a positive correlation between α-SMA content and disease recurrence (high: 58.1%; low: 11.9%; χ^2^ test, *p <* 0.001). In addition, a higher α-SMA expression in the largest MLNs predicated lower RFS (log-rank *p <* 0.001) and OS (log-rank *p =* 0.001); Figure 3A. We conducted a subgroup analysis with Stage III patients and found a similar correlation of α-SMA expression with RFS (log-rank *p* < 0.001) and OS (log-rank *p <* 0.001); Figure 3B.

At the invasive front of PTs (all patients), high α-SMA expression correlated with shorter OS, but not with RFS (Supplementary Figure 5A). Subgroup analysis showed that in Stage III patients, α-SMA expression at the invasive front of the PTs did not significantly correlate with OS or RFS (Supplementary Figure 5B).

### Univariate and multivariate Cox proportional hazards regression analyses to identify the prognostic factors associated with OS

CAFs produce transforming growth factor beta (TGF-β) and promote the expansion of extracellular matrix including type I collagen. In other words, both CAF and collagen are indicators of fibrosis, and their expression levels are expected to be correlated in cancer tissues. There was moderate positive correlation between α-SMA expression and collagen deposition rate in PTs (Pearson’s correlation coefficient: 0.521; Supplementary Figure 6A) and in MLNs (Pearson’s correlation coefficient: 0.603; Supplementary Figure 6B). Therefore, since the staining rates of α-SMA and collagen are confounding factors, these were analyzed separately.

We carried out univariate and multivariate Cox proportional hazards regression analyses to identify possible prognostic factors for OS in all patients (Table 2) and in Stage III patients (Table 3). Univariate Cox regression analysis showed that pathological tumor stage, pathological node stage, preoperative CEA, liver metastasis, peritoneal dissemination, and α-SMA expression in PTs and MLNs correlated significantly with OS. Multivariate Cox regression analysis showed that liver metastasis (HR, 1.80; 95% CI, 1.56–8.28; *p =* 0.005), peritoneal dissemination (HR, 1.96; 95% CI, 1.39–15.37; *p =* 0.033), and α-SMA expression in MLNs (HR, 1.53; 95% CI, 1.03–4.94; *p =* 0.034) were independent prognostic factors for OS in all patients (Table 2). Expression rate of α-SMA in MLNs remained as a predictive factor for OS, when data from all patients as well as for Stage III patients were analyzed (HR, 3.01; 95% CI, 1.54–6.60; *p =* 0.006; Table 3).

**Table 2:** Univariate and multivariate Cox proportional hazards regression analyses of clinicopathological factors including α-SMA expression rate in primary tumors and metastatic lymph nodes for overall survival in Stage III/IV colorectal cancer patients

Factors		Univariate			Multivariate		
		HR	95% CI	*p*-value	HR	95% CI	*p*-value
Pathological tumor stage	T2,3/T4	3.55	1.72–7.29	0.001^*^	1.45	0.18–11.39	0.391
Pathological node stage	N1/N2	1.65	1.17–2.34	0.005^*^	1.34	0.77–3.50	0.145
Preoperative CEA	≦5/>5	1.95	1.21–3.14	0.006^*^	1.37	0.85–10.44	0.233
Liver metastasis	H–/H+	2.26	1.60–3.20	<0.001^*^	1.80	1.56–8.28	0.005^*^
Peritoneal dissemination	P–/P+	1.94	1.14–3.29	0.015^*^	1.96	1.39–15.37	0.033^*^
α-SMA in primary tumors	Low/High	1.56	1.04–2.33	0.030^*^	1.18	0.56–3.55	0.463
α-SMA in metastatic lymph nodes	Low/High	1.80	1.25–2.59	0.002^*^	1.53	1.03–4.94	0.034^*^

^*^Statistically significant. Abbreviations: α-SMA: α-smooth muscle actin; CEA: Carcinoembryonic antigen; HR: hazard ratio; CI: confidence interval.

**Table 3:** Univariate and multivariate Cox proportional hazards regression analyses of clinicopathological factors including α-SMA expression rate in primary tumors and metastatic lymph nodes for overall survival in only Stage III colorectal cancer patients

Factors		Univariate			Multivariate		
		HR	95% CI	*p*-value	HR	95% CI	*p*-value
Pathological tumor stage	T2,3/T4	3.20	0.85–12.11	0.086	1.21	0.23–6.34	0.824
Pathological node stage	N1/N2	1.84	1.01–3.33	0.045^*^	1.54	0.76–3.12	0.233
Preoperative CEA	≦5/>5	1.33	0.72–2.46	0.360	1.27	0.67–2.39	0.467
α-SMA in primary tumors	Low/High	1.10	0.61–2.00	0.747	1.17	0.64–2.15	0.608
α-SMA in metastatic lymph nodes	Low/High	3.17	1.47–6.85	0.003^*^	3.01	1.54–6.60	0.006^*^

^*^Statistically significant. Abbreviations: α-SMA: α-smooth muscle actin; CEA: Carcinoembryonic antigen; HR: hazard ratio; CI: confidence interval.

Then, we performed Cox regression analyses by adding collagen deposition as a factor instead of α-SMA expression. Collagen deposition in PTs and MLNs correlated significantly with OS in Stages III / IV univariate analysis. However, collagen deposition rates of PTs and MLNs were not independent prognostic factors in multivariate analysis (Supplementary Table 1). Collagen deposition rates in PTs and MLNs were not prognostic predictors even in Stage III multivariate analysis (Supplementary Table 2).

On the other hand, higher α-SMA expression and collagen deposition in primary tumors were associated with short OS, but they were not significant factors in multivariate Cox regression analyses.

### α-SMA positive stromal cells in MLN are fibroblastic reticular cells (FRCs) that co-express podoplanin (PDPN)

**Figure 4:**
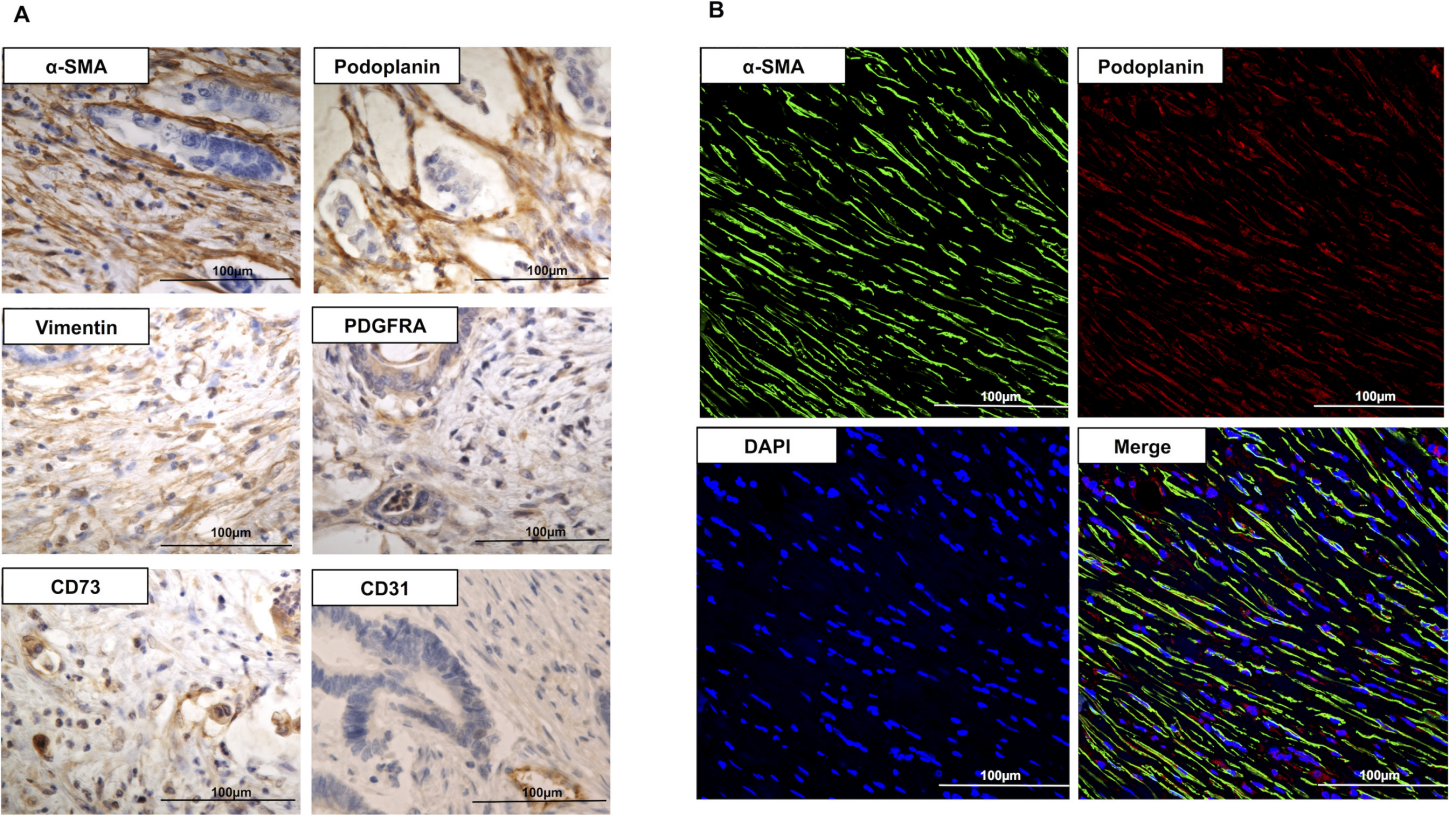
(**A**) Immunohistochemistry for α-SMA, PDPN, vimentin, PDGFRA, CD73, and CD31 in metastatic lymph node (400×). α-SMA, PDPN, vimentin, PDGFRA, and CD73 stained stromal cells in metastatic lymph node. CD31 was not stained in stromal cells. (**B**) Immunofluorescence images of the stroma of a metastatic lymph node (400×), stained for α-SMA (green), PDPN (red), and DAPI (blue). Dual staining for PDPN and α-SMA confirms the co-expression of the two proteins in the FRCs of metastatic lymph node. α-SMA: α-smooth muscle actin; PDPN: podoplanin; PDGFRA: *platelet-derived growth factor receptor α;* DAPI: 4’,6-Diamidino-2-phenylindole; FRCs: fibroblastic reticular cells.

We performed IHC staining of stromal cells in MLNs with α-SMA, vimentin, and CD73 (a marker of mesenchymal cells). In order to confirm that the stromal cells are FRC, we performed immunostaining for positive markers: PDPN, *platelet-derived growth factor receptor alpha* (PDGFRA); and negative marker: CD31 (Figure 4A). We confirmed that α-SMA, PDPN, vimentin, PDGFRA, and CD73 were expressed in stromal cells of MLNs. On the other hand, CD31 stained vascular endothelial cells, but other stromal cells were not stained.

In 95% of patients (38/40 patients) with high MLN α-SMA expression, the stromal tissues of MLNs were stained positively for PDPN. Furthermore, the PDPN signal co-localized with α-SMA expression, which was further confirmed by IHC dual staining (Figure 4B); implying that the stromal cells in MLNs were FRCs.

## DISCUSSION

We demonstrated in this study that the high α-SMA expression in MLNs is a strong predictive factor for recurrence and survival rate among patients with advanced CRC. Our study demonstrates the clinical impact of fibrosis in MLNs on CRC.

Neoplastic cells may stimulate tumor stromal fibroblasts via TGF-β pathway to cause tissue fibrosis and matrix production [12, 19, 20], resulting in a desmoplastic reaction (DR). Recent clinical reports hypothesize that stromal fibrosis of PTs promotes invasion and metastasis in CRC, and that DR contributes to poor prognosis [21, 22]. Tsujino [13] reported that the abundance of tumor myofibroblasts predicted shorter disease-free survival in Stages II and III CRC. Halvorsen [14] reported that pronounced fibrosis at the tumor edge correlates with unfavorable stage distributions, while Herrera [15] reported that the presence of tumor CAFs dictates clinical outcome in CRC patients, implying that fibrosis in the PT accelerates tumor progression. As in these reports, we investigated α-SMA expression in both the central part and the invasive front of PTs and revealed that stromal fibrosis at the invasive front, not the central part, of PTs was correlated with poor prognosis (data not shown). We speculated that the difference in clinical meaning between tumor invasive front and tumor central occurred because of the hypoxic condition and high interstitial pressure. These severe conditions might cause apoptosis of fibroblasts and change in fibrotic condition in the central part of the PT. We suggested that this difference in the microenvironment caused the fibrotic status. It is known that cancer cells at the invasive front of PT stimulate TGF-β production and increases the extracellular matrix, including collagen [23], resulting in the induction of epithelial-mesenchymal transition followed by the promotion of tumor invasion and metastasis [24]. In contrast to the above, Özdemir *et al.* [16] showed that the suppression of SR or removal of fibroblasts around the PTs in mice pancreatic cancer correlated with shorter survival. Thus, the effect of peri-tumoral stromal response on tumor progression is still controversial.

Several multicenter prospective CRC studies revealed that only the pathological stage (specified as pathological tumor stage, pathological node stage, and distant metastasis) was a reliable prognostic factor [25, 26]. In addition, intestinal perforation or obstruction, residual cancer cells in the surgical margin, lymphatic permeation, perineural invasion, poorly differentiated histology, and high preoperative CEA level also signify worse prognosis [27–29]. Consistent with the above, we showed that pathological tumor stage, pathological node stage, liver metastasis, peritoneal dissemination, and preoperative CEA in univariate analysis, and liver metastasis and peritoneal dissemination in multivariate analysis, significantly influenced disease prognosis. Surprisingly, the fibrosis of the largest MLNs reflected poor prognosis strongly same as the above factors, implying that fibrosis in MLNs may be a promising biomarker.

The tumor microenvironment hosts diverse cell types, including immune cells, fibroblasts, and endothelial cells, which dynamically remodel tumor tissue [12, 30, 31]. Fibroblasts, localized in tumor stromal tissue (CAFs) express α-SMA [12, 32, 33], which is an actin 6 isoform, important for contractile function [34]. Tissue fibrosis facilitates cell-to-cell interactions, which play a major role in carcinogenesis [35–37]. Initial host-tumor interactions activate wound-repair mechanisms and scar-tissue formation around the tumor. However, subsequently, malignant cells facilitate growth factor release, leading to neovascularization and tumor invasion. One hypothesis states that the dense collagen secreted by myofibroblasts represents an attempt to defend the host by containing the tumor and delaying vascularization [38]. CAFs may originate from resident fibroblasts, mesenchymal stem cells, fibrocytes, or transformed endothelial cells by epithelial-mesenchymal transition [39].

Tumor metastasis is a factor of poor prognosis. In this study, the multivariate Cox analysis also revealed that liver metastasis was a crucial poor prognosis. The aim of this study was to demonstrate whether or not angiogenesis or lymphangiogenesis, which facilitates tumor metastasis at the invasive front of PT, is associated with stromal fibrosis. Therefore, for the PT, we stained CD31, the marker for blood vessels, and PDPN, the marker for lymphatic vessels, and investigated correlations between the expression rates of these markers at the invasive front of PT and those of α-SMA and collagen (Pearson’s correlation coefficient: CD31 and α-SMA, 0.109; PDPN and α-SMA, 0.168). According to our findings, associations between these factors appeared weak. It is also possible that fibrosis which induces tumor growth and metastasis, are the secondary effects of increased tumor growth. Fibrotic change may also physically facilitate the migration of tumor cells. In addition, we also stained Ki67 to investigate the relationship between fibrosis in the tumor and tumor cell proliferation (data not shown). There was no correlation between expression of Ki67 and those of α-SMA and collagen (Pearson’s correlation coefficient: Ki67 and α-SMA, 0.028). Thus, no correlation was shown between tumor metastasis stimulating factors, such as angiogenesis, lymphangiogenesis and tumor cell growth in PT; and fibrosis of tumor-associated stroma. This suggests that fibrotic change is not facilitated by the secondary effect of the tumor metastasis stimulating factors.

From past study, it is known that the stromal fibroblastic cells in LNs are FRCs. In the paracortical areas of the LNs, FRCs are the structural skeleton of the LN and express PDPN, producing collagen III rich reticular fibers that form a dense network within the lymphoid tissue [40]. FRCs are immunologically specialized myofibroblasts of mesenchymal origin. They can be differentiated from other lymph node-resident cells by the expression of PDPN and PDGFRA, and the lack of expression of CD31 and CD45. FRCs express molecules common to many myofibroblasts, including α-SMA, vimentin, desmin, CD90, CD73, CD103, and the ERTR7 antigen [41]. PDPN is known to increase the migratory ability of dendritic cells and induce angiogenesis by acting on platelets in the medulla of LNs. FRCs recognize bacteria, viruses, and other microorganisms; thus preventing infection [42–47]. Here, we show that the stroma of MLNs contains α-SMA positive cells with PDPN expression, suggesting that the mesenchymal cells in MLNs may be FRCs. Though Yeung *et al.* [48] reported the presence of myofibroblasts in MLNs of CRC patients, we proved for the first time that a part of myofibroblasts in MLN are FRCs.

It is novel to determine whether or not fibrotic change in metastatic lymph nodes, as a marker, can determine whether a patient should receive either adjuvant chemotherapy or any other drug. According to the results of our study, no difference was observed in OS and RFS regardless of whether the patient had received either adjuvant chemotherapy or oxaliplatin (out of all chemotherapy drugs), or not; both in all patients and in Stage III patients. We also categorized the patients into two (severe or mild fibrosis groups), and then investigated their survival rates with or without adjuvant chemotherapy. However, there was no significant difference observed between the two groups (data not shown). The subjects in this study were in Stages III or IV. More than 80% of the patients received adjuvant chemotherapy while patients in poor general condition or the elderly had not received chemotherapy; suggesting that the patients’ background, whether or not they had received chemotherapy varied greatly. Therefore, there is a limitation in this retrospective study. For further investigation, a prospective cohort study is suggested with a larger sample size.

In summary, we show that fibrosis in MLNs is a strong prognosticator of poor OS and RFS in CRC patients. Malignant cells with high metastatic potential may be prone to causing fibrotic reactions and collagen deposition in LNs. We also showed that patients with fibrotic LN metastases are at high risk of recurrence and shorter survival rate. Intensive follow up is therefore recommended in such patients.

The primary limitation of this study is its retrospective, single-center nature. In addition, the sample size was small, and the role of FRCs was not clarified. To further understand the clinical implications of metastatic fibrotic LNs in CRC patients, a prospective study with a larger sample size and long-term follow-up is required. Under the present circumstances, the role of α-SMA expressing FRC in MLNs was not clarified. By elucidating its mechanism, the therapeutic benefit of the influence of stromal response can be assessed.

## MATERIALS AND METHODS

### Patients and samples

We reviewed a total of 104 consecutive colorectal adenocarcinoma patients with LN metastasis who underwent colectomy between January 2010 and December 2013 at Shiga University of Medical Science Hospital (Shiga, Japan). We excluded three patients who had other carcinomas (i.e., liver, lungs, or ovaries) and seven, who received oxaliplatin-based preoperative chemotherapy from the analyses. Finally, a total of 94 patients were included in the analysis. The tissue samples included 66 Stage III and 28 Stage IV carcinomas; graded according to the Union for International Cancer Control *(UICC)* tumor-node-metastasis (TNM) classification of malignant tumors, 7th edition [49]. Based on the Japanese Society for Cancer of Colon and Rectum (JSCCR) guidelines [50], the majority of patients (76 [80.9%]) received 5-fluorouracil-based postoperative chemotherapy (47 of 76 patients received oxaliplatin-added chemotherapy while the remaining 29, received oxaliplatin-free chemotherapy). The follow-up protocol in CRC survivors involved physical examination and tumor marker tests, every three months, as well as CT scan, every six months. Tumor samples were classified into “High” and “Low” staining rate groups. This research was approved by the Human Ethics Review Committee of Shiga University of Medical Science. We published an opt-out option on the Website of Shiga University of Medical Science; hence, the requirement for written informed consent was waived.

### H&E staining and IHC

Tissue sections (4 µm thick) were prepared from 10% formalin-fixed paraffin-embedded blocks and stained with H&E. For IHC, slides were deparaffinized by xylene treatment and rehydrated by passing them through an ethanol gradient, and then heated in an electric kettle with antigen retrieval solution (Immunosaver^®^, Nisshin EM, Tokyo, Japan). Endogenous peroxidases were inactivated in a methanolic 3% hydrogen peroxide solution for 10 minutes, and subsequently incubated with a blocking reagent *(*Blocking One^®^, Nacalai Tesque, Kyoto, Japan) for 20 minutes. The tissue sections were incubated overnight at 4°C with an anti-α-SMA antibody (1:100, ab7817, Abcam, Cambridge, UK), anti-PDPN antibody D2-40 (code 413451, NICHIREI BIOSCIENCES INC., Tokyo, Japan), anti-vimentin antibody (1:100, M7020, Dako, Carpinteria, USA), anti-fibronectin antibody (1:100, ab2413, Abcam), anti-FAP antibody (1:100, ab28244, Abcam), anti-PDGFRA antibody (1:200, #5241, Cell Signaling Technology, Inc., Danvers, USA), anti-CD73 antibody (1:100, ab133582, Abcam), anti-CD31 antibody (1:50, ab124432, Abcam), or anti-Ki67 antibody (1:100, ab16667, Abcam). The following day, the slides were incubated with a secondary antibody (Simple Stain MAX PO^®^, NICHIREI BIOSCIENCES INC.) for 30 minutes, and the antigen was visualized by diaminobenzine staining (DAB^®^, DAKO) for 10 minutes.

### Collagen staining

Collagen (type I and type III) was stained with Picro-Sirius Red, using the Elastica van Gieson staining kit (MUTO PURE CHEMICALS, Tokyo, Japan). Tissue sections (4 µm thick) were incubated for 1 hour with a resorcin-fuchsin solution and further incubated for 5 minutes with Weigert’s iron hematoxylin solution. A final incubation was performed with 1% Sirius Red and van Gieson’s stain for 10 minutes. Collagen (type I and type III) was stained red by Sirius Red.

### Dual immunofluorescence staining

Tissue sections (2 µm thick) were processed using the IHC protocol as above. PDPN was detected with anti-PDPN antibody (1:200, bs-17742R, Bioss Inc., Massachusetts, USA), and α-SMA with an anti-α-SMA antibody (1:100, ab7817, Abcam). Secondary antibodies employed were Alexa Fluor 488 goat anti-mouse (A-11029), or Alexa 594 goat anti-rabbit (A-11012) (Molecular Probes, Invitrogen, Carlsbad, USA), at a dilution of 1:200. Tissues were incubated with secondary antibodies for 60 minutes. ProLong^®^ Diamond Antifade Mountant with 4’,6-diamidino-2-phenylindole (P36962, Molecular probes, Invitrogen) was used as the mounting agent. A Fluoview FV1000-D microscope (Olympus, Tokyo, Japan) was used for image analysis.

### IHC and collagen scoring

Blinded microscopic evaluation of the slides was performed by an experienced pathologist (K.M.). The pathologist, in consultation with the author (D.I.), demarcated five areas at a magnification of 200× for analyses, and the average staining rate of stromal cells excluding tumor cells was quantitated by Image J (National Institutes of Health, Bethesda, USA).

We examined the largest cross-sectional slice in PTs, emphasizing the invasive front (features here reflect tumor metastatic potential) [24, 25]. The muscle layer was excluded from the assessment, since the muscle fibers are known to express α-SMA. We used the staining rate of the largest diameter MLNs for the analyses of survival rate. The expression of α-SMA and Collagen in non-MLN stroma was very few and there was no difference in their expression between non-MLNs of a single patient. Therefore, we used the largest diameter non-MLN for analysis.

### Statistical analysis

Statistical analysis was performed using the Statistical Package for the Social Sciences software (SPSS, version 22.0, IBM, Armonk, New York, USA). RFS was the time from curative surgery to the time of first tumor recurrence or the final follow-up date while OS was the time from colorectal cancer resection to the time of all-cause mortality or the final follow-up date. Survival analysis was by the Kaplan–Meier method, and the comparison of survival time between subgroups was by log-rank test. Multivariate analysis was performed using the Cox proportional hazards regression model. Associations between categorical variables were analyzed using either the χ^2^ test or the Fisher’s exact test. We correlated the data based on the tumor categories with the clinicopathological factors. The staining rates of α-SMA and collagen in all specimens followed the normal distribution by Kolmogorov-Smirnov test. Mean values were reported from the Student’s *t*-test and expressed as the mean value ± SD when data followed a normal distribution. We used the Mann–Whitney *U* test to compare factors (age and preoperative CEA) that did not follow the normal distribution. The receiver operating characteristic (ROC) curve analyses based on the 48-month OS of all patients (Supplementary Figure 7A–7D) was performed; while the maximum value of the Youden’s Index (Sensitivity + Specificity – 1) was determined as the cut-off value of α-SMA expression and the collagen deposition rate. We determined the “High” and “Low” staining rate groups as cases with higher and lower staining rates, respectively, compared to the cut-off value. The same cut-off value used for analyses of OS in all patients was also used for RFS and Stage III patients’ analyses. The significance level was set at *p <* 0.05.

## SUPPLEMENTARY MATERIALS FIGURES AND TABLES


