# Supplementary material for: Fibrosis in metastatic lymph nodes is clinically correlated to poor prognosis in colorectal cancer
Source: Oncotarget. 2018 Jul 3;9(51):29574–86. doi: 10.18632/oncotarget.25636 (PMC6049853; doi:10.18632/oncotarget.25636)
Supplement: Supplementary file 1 [file oncotarget-09-29574-s001.pdf]

Fibrosis in metastatic lymph nodes is clinically correlated to poor prognosis in colorectal cancer

SUPPLEMENTARY MATERIALS

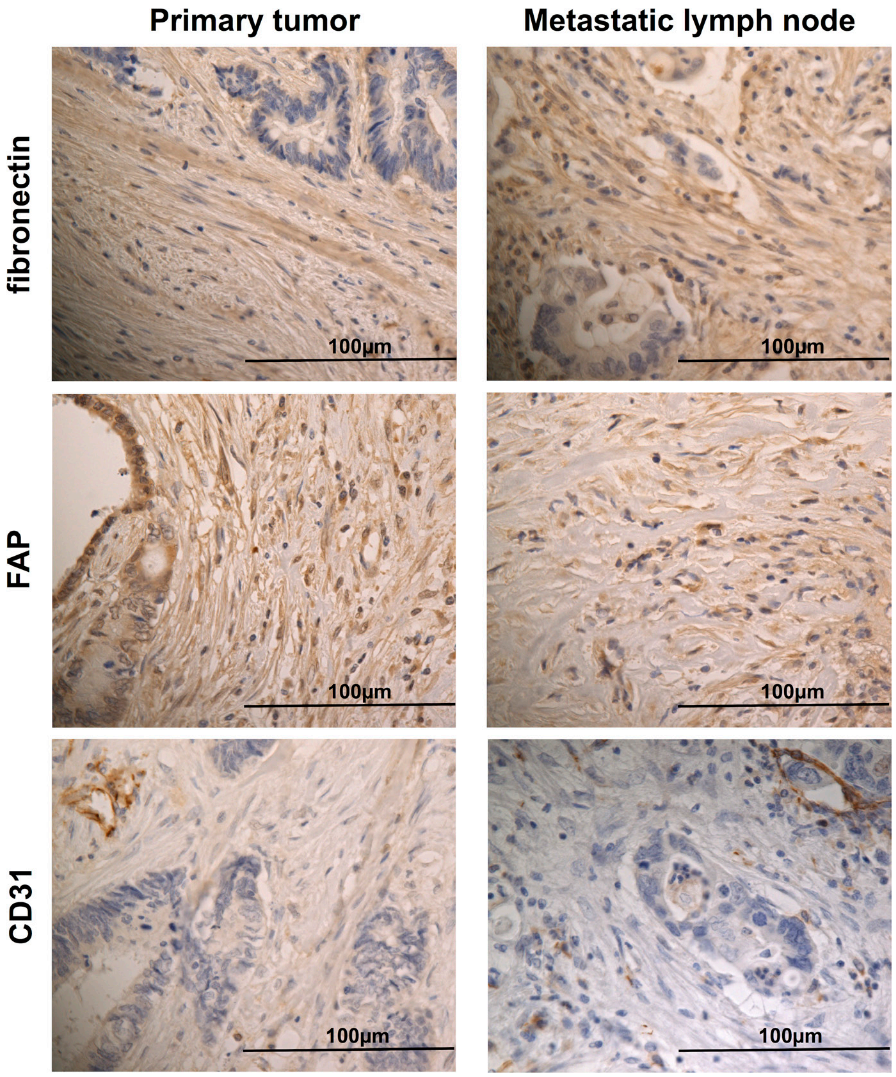

Supplementary Figure 1: Immunohistochemistry staining of fibronectin, FAP, and CD31 in primary tumor and metastatic lymph node (400×). Fibronectin and FAP stained stromal cells in both tissues. CD31 was not stained in stromal cells. FAP: fibroblast activation protein.

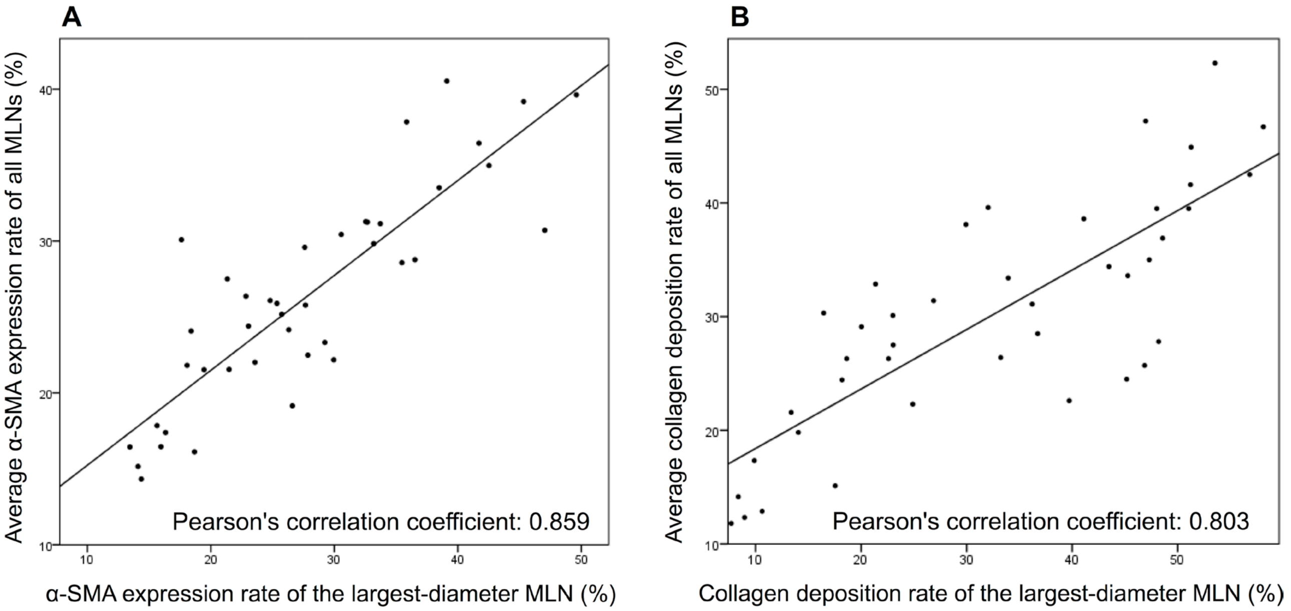

Supplementary Figure 2: Scatter diagram comparing (**A**) α-SMA expression or (**B**) collagen deposition rate of the largest-diameter MLN and the average expression rate of all MLNs in the same patient (line represents the regression line). α-SMA: α-smooth muscle actin; MLN: metastatic lymph node.

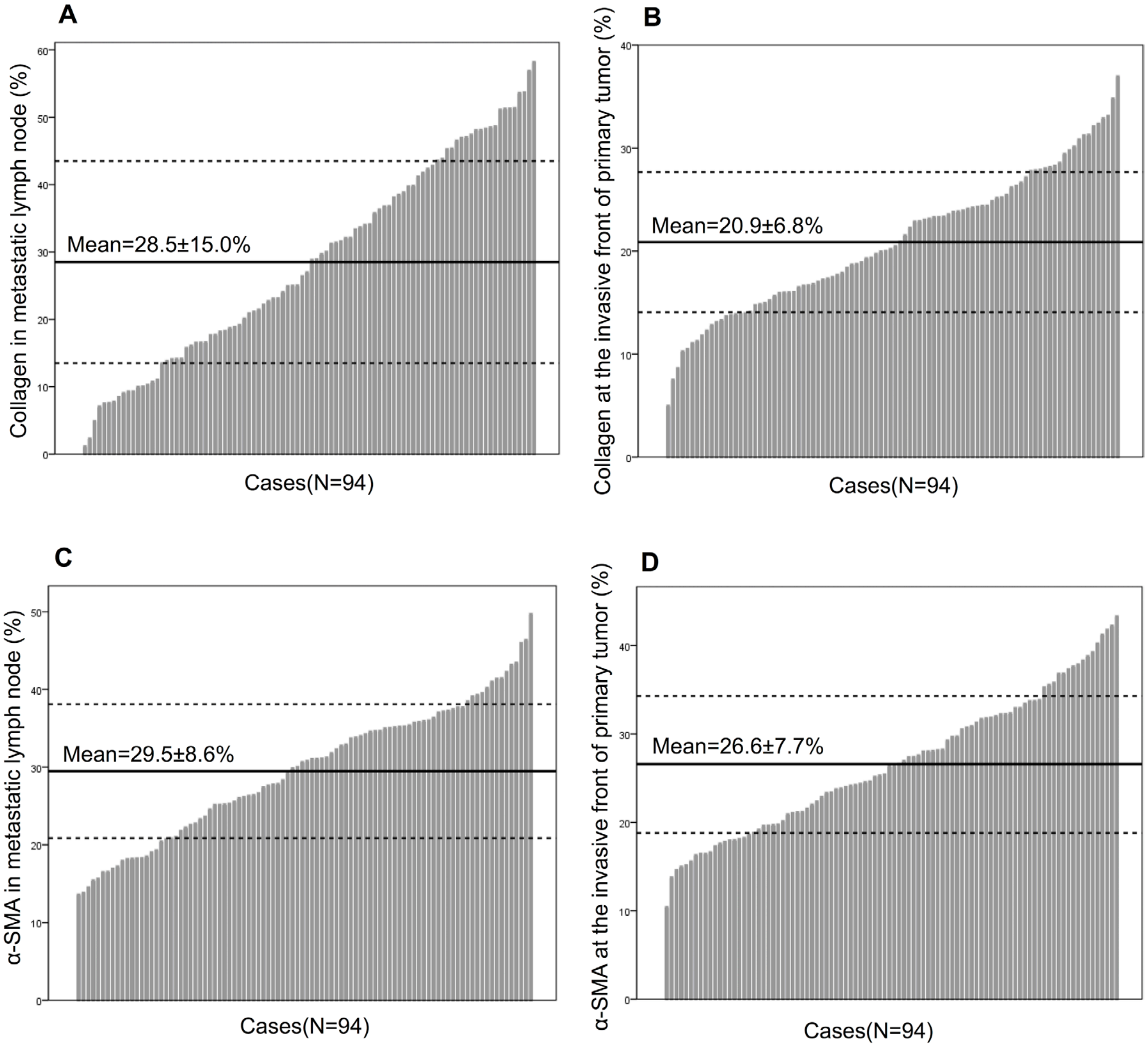

Supplementary Figure 3: Distribution of collagen deposition rate (**A**) in metastatic lymph nodes and (**B**) at the invasive front of primary tumors, and α-SMA expression rate (**C**) in metastatic lymph nodes and (**D**) at the invasive front of primary tumors (dotted lines represent ± standard deviation [SD] range). α-SMA: α-smooth muscle actin.

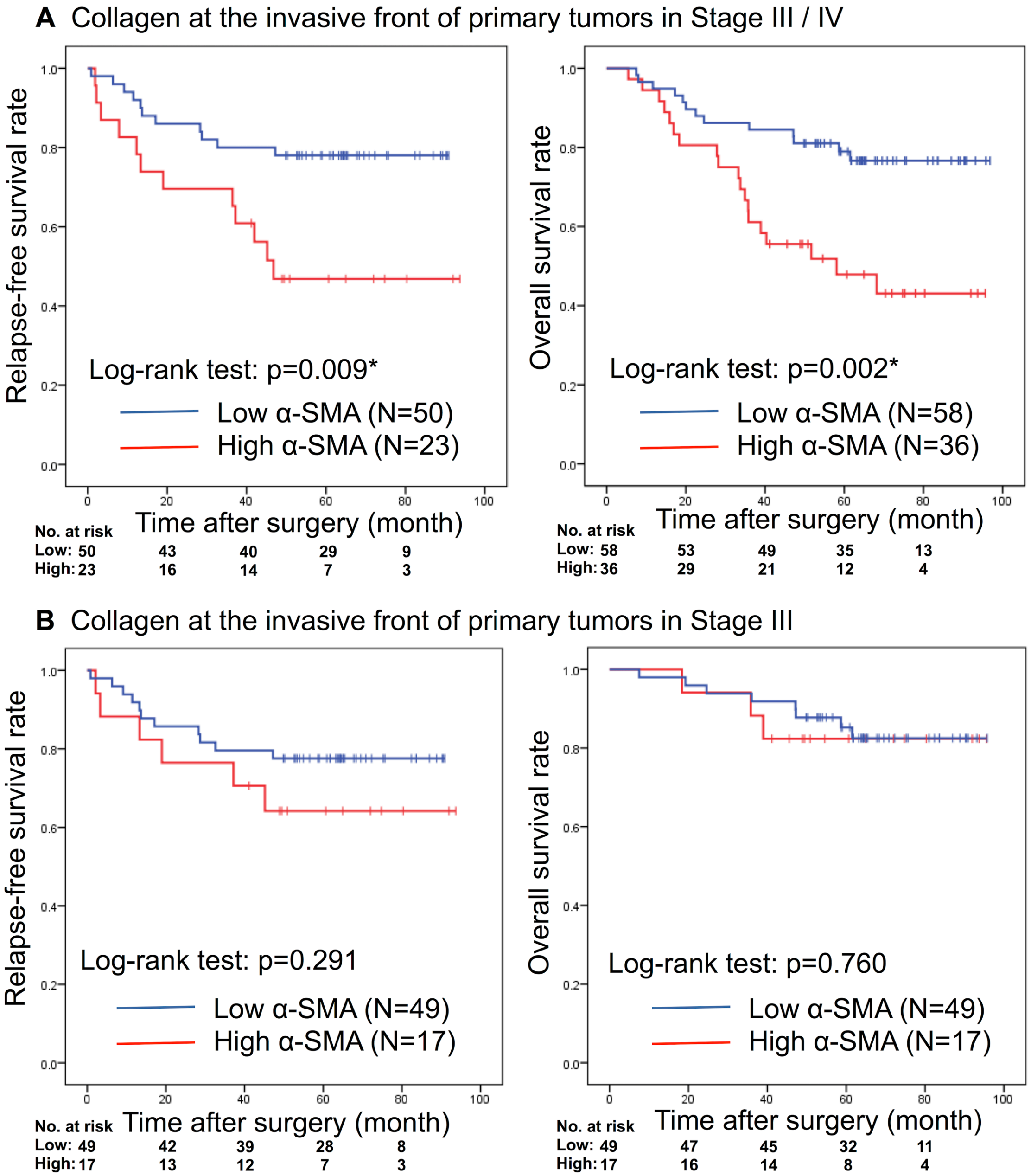

Supplementary Figure 4: Kaplan–Meier analyses of RFS and OS according to the collagen deposition rate at the invasive front of primary tumors in (**A**) Stages III / IV disease or (**B**) only Stage III disease. RFS: relapse-free survival; OS: overall survival. *Statistically significant.

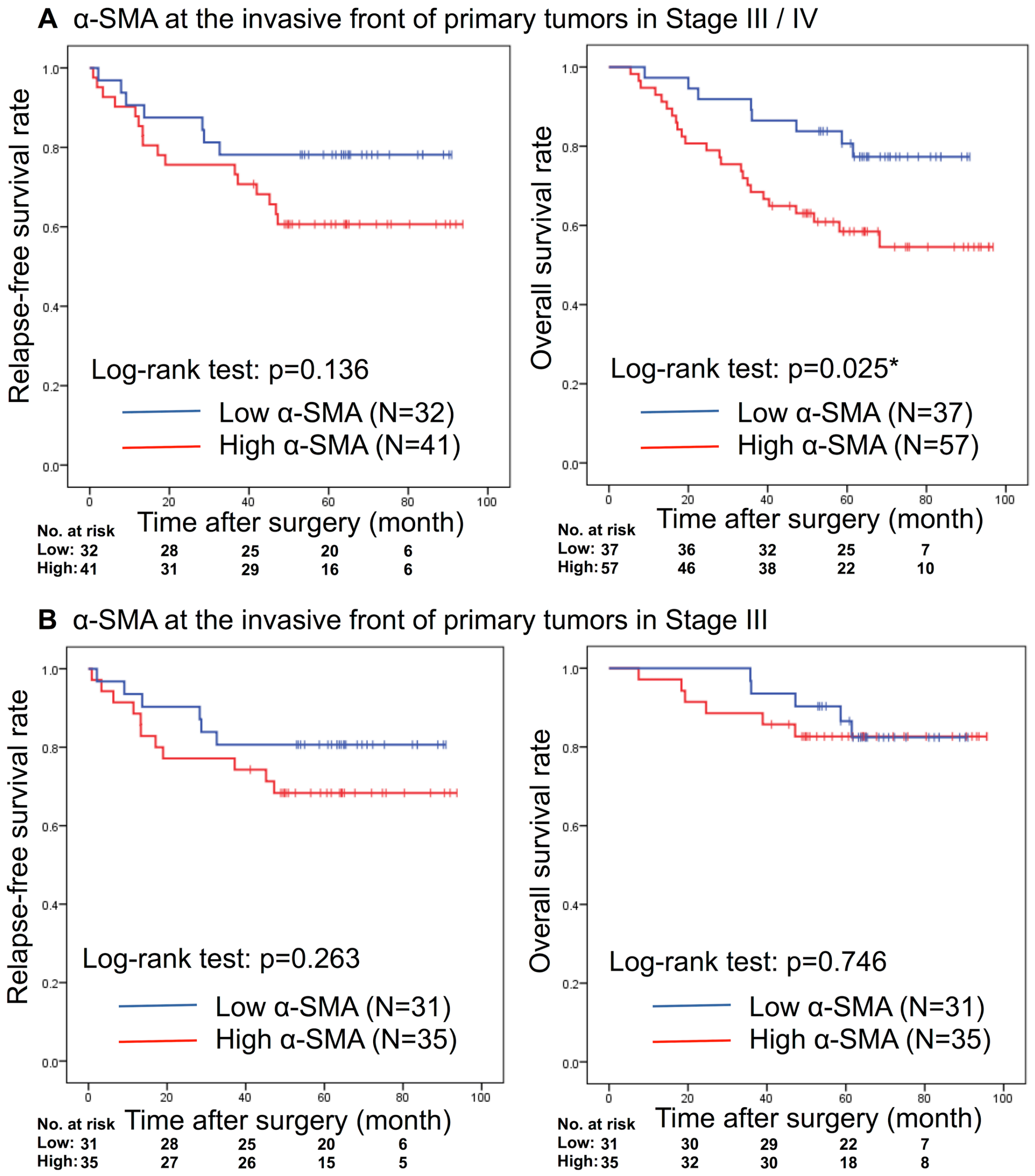

Supplementary Figure 5: Kaplan–Meier analyses of RFS and OS according to α-SMA expression rate at the invasive front of primary tumors in (**A**) Stages III/IV disease or (**B**) only Stage III disease. RFS: relapse-free survival; OS: overall survival; α-SMA: α-smooth muscle actin. *Statistically significant.

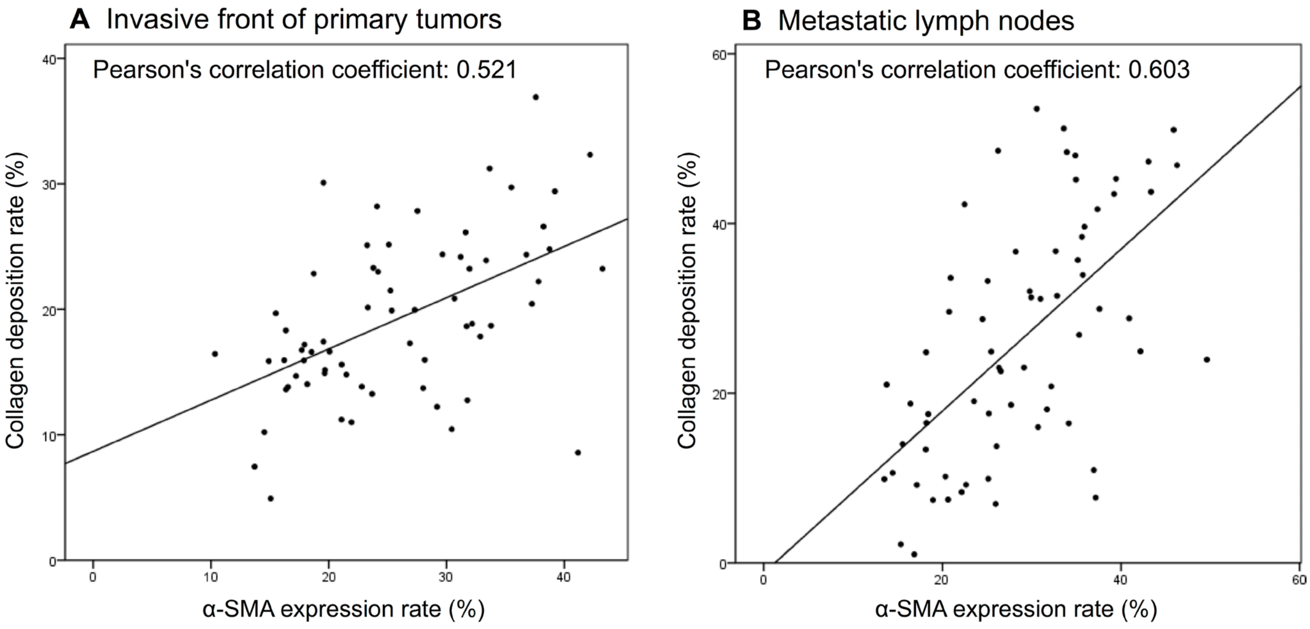

Supplementary Figure 6: Scatter diagrams comparing the relationship between α-SMA expression and collagen deposition rate (**A**) at the invasive front of primary tumors and (**B**) in metastatic lymph nodes (line represents the regression line). α-SMA: α-smooth muscle actin.

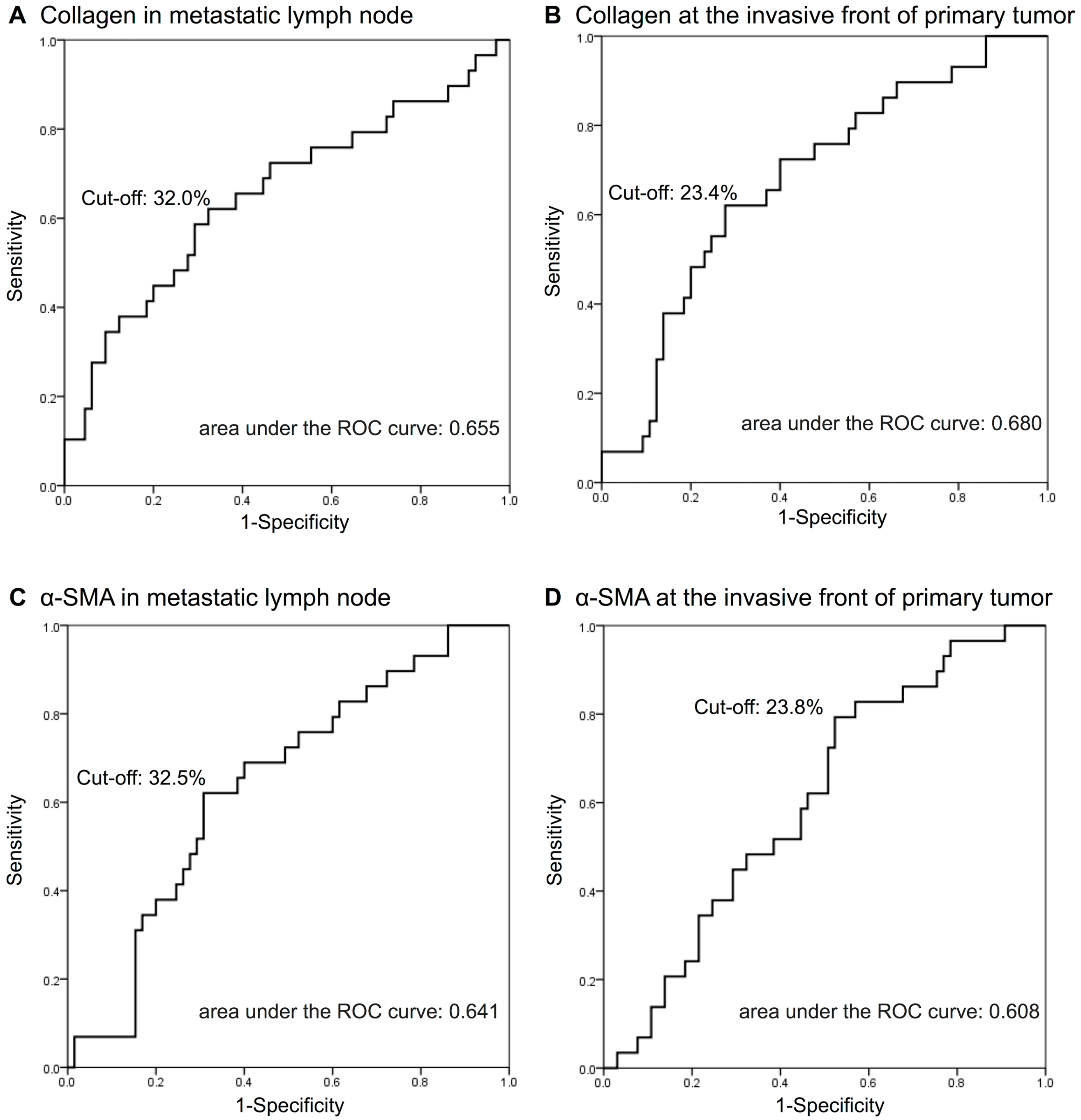

Supplementary Figure 7: ROC curve analyses for 48-month OS with collagen deposition rate (**A**) in metastatic lymph nodes and (**B**) at the invasive front of primary tumors; and α-SMA expression rate (**C**) in metastatic lymph nodes and (**D**) at the invasive front of primary tumors. Cut-off values for collagen and α-SMA were based on the ROC curves. α-SMA: α-smooth muscle actin; ROC curve: receiver operating characteristic curve; OS: overall survival.

Supplementary Table 1: Univariate and multivariate Cox proportional hazards regression analyses of clinicopathological factors including collagen deposition rate in primary tumors and metastatic lymph nodes for overall survival in Stage III/IV colorectal cancer patients

Factors		Univariate			Multivariate		
		HR	95% CI	*p*-value	HR	95% CI	*p*-value
Pathological tumor stage	T2,3/T4	3.55	1.72–7.29	0.001*	1.73	0.77–3.91	0.186
Pathological node stage	N1/N2	1.65	1.17–2.34	0.005*	1.71	0.79–3.73	0.174
Preoperative CEA	≦5/>5	1.95	1.21–3.14	0.006*	1.66	0.58–4.75	0.347
Liver metastasis	H–/H+	2.26	1.60–3.20	<0.001*	3.53	1.54–8.10	0.003*
Peritoneal dissemination	P–/P+	1.94	1.14–3.29	0.015*	2.78	0.84–9.29	0.096
Collagen in primary tumors	Low/High	1.72	1.21–2.45	0.003*	1.13	0.47–2.77	0.783
Collagen in metastatic lymph nodes	Low/High	1.79	1.24–2.58	0.002*	1.74	0.78–3.88	0.180

*Statistically significant. CEA: Carcinoembryonic antigen; HR: hazard ratio; CI: confidence interval.

Supplementary Table 2: Univariate and multivariate Cox proportional hazards regression analyses of clinicopathological factors including collagen deposition rate in primary tumors and metastatic lymph nodes for overall survival in only Stage III colorectal cancer patients

Factors		Univariate			Multivariate		
		HR	95% CI	*p*-value	HR	95% CI	*p*-value
Pathological tumor stage	T2,3/T4	3.20	0.85–12.11	0.086	1.63	0.31–8.41	0.562
Pathological node stage	N1/N2	1.84	1.01–3.33	0.045*	2.20	0.56–8.68	0.262
Preoperative CEA	≦5/>5	1.33	0.72–2.46	0.360	1.24	0.34–4.51	0.747
Collagen in primary tumors	Low/High	1.11	0.57–2.16	0.760	1.20	0.28–5.09	0.809
Collagen in metastatic lymph nodes	Low/High	2.03	1.10–3.76	0.024*	2.96	0.79–11.09	0.107

*Statistically significant. CEA: Carcinoembryonic antigen; HR: hazard ratio; CI: confidence interval.
